# Facile minocycline deployment in gingiva using a dissolvable microneedle patch for the adjunctive treatment of periodontal disease

**DOI:** 10.1002/btm2.10730

**Published:** 2024-10-20

**Authors:** Huimin Li, Xueyu Wen, Xinyi Gong, Yange Wu, Puxuan Zhao, Yun Zhang, Zhuomin Sha, Hao Chang, Xuepeng Chen

**Affiliations:** ^1^ School of Stomatology, Zhejiang University School of Medicine, Clinical Research Center for Oral Diseases of Zhejiang Province, Key Laboratory of Oral Biomedical Research of Zhejiang Province, Cancer Center of Zhejiang University, Stomatology Hospital Hangzhou Zhejiang China; ^2^ Hangzhou Institute of Medicine, Chinese Academy of Sciences Hangzhou Zhejiang China

**Keywords:** dissolvable microneedles, gingival delivery, inflammation modulation, minocycline, periodontitis

## Abstract

Minocycline is a commonly used drug for adjunctive therapy in periodontal disease. However, the current mainstream local medications primarily rely on intra‐pocket administration, which, while avoiding the side effects of traditional systemic drugs, presents challenges such as inconvenience, discomfort, and the need for professional assistance, thus affecting patient compliance. Herein, we introduce a minocycline‐loaded dissolvable microneedle (Mino‐DMN) patch that allows for local and efficient delivery of minocycline to gingiva for the treatment of periodontitis. A two‐step casting micro‐molding process involving vacuum drying and freeze drying is employed to concentrate minocycline in the microneedle part and limit its diffusion into the patch backing. The resulting Mino‐DMN patch features an array of minocycline‐enriched gelatin MNs with a porous HA patch backing. The microneedles can penetrate into gingiva with enough mechanical strength and quickly release minocycline into the gingival tissue, ensuring prolonged local residence of the drug and minimizing its loss to saliva. In vivo experiments show Mino‐DMN inhibits pro‐inflammatory factors, promotes anti‐inflammatory factors, and stimulates bone formation, surpassing topical application and comparable to the inconvenient and discomfort administration of Periocline®. This proposed Mino‐DMN offers a simple, efficient, user‐friendly strategy for the adjunctive treatment of periodontal disease.

**Translational Impact Statement**

Plaque control stands as a crucial measure in the treatment of periodontal disease, with local medications serving as pivotal adjunctive tools. In this study, we have devised a DMN patch for localized minocycline delivery for periodontitis treatment. Experimental evidence underscores the ease with which the DMN can deliver minocycline, thereby eliciting antimicrobial and anti‐inflammatory effects. The clinical translation of these findings holds promise in furnishing periodontal disease patients with a more comfortable and convenient treatment modality, thereby fostering enhanced patient compliance and facilitating self‐management.

## 1 INTRODUCTION

Periodontitis, a chronic inflammatory oral condition, is characterized by gingival infections and damage of supporting tissues like periodontal membrane and alveolar bone,^1^, ^2^ eventually resulting in tooth mobility and loss.^3^, ^4^, ^5^ Notably, periodontitis is associated with systemic health issues like cardiopathy,^6^, ^7^ diabetes,^8^, ^9^ and hypertension,^10^, ^11^ causing a huge social and economic burden. Periodontitis arises from an imbalance between subgingival bacterial plaque and the host's immune response.^12^ In the first place, it is initiated by bacterial infection, with a complex subgingival plaque composition, including Gram‐negative anaerobic bacteria like *Porphyromonas gingivalis*.^13^, ^14^ Additionally, bacteria can stimulate host immune‐inflammatory responses, contributing to tissue damage.^15^, ^16^, ^17^ In periodontitis patients, tissue destruction leads to pathological deepening of gingival crevices, forming periodontal pockets. These pockets lack the flushing action of saliva and present physical limitations for effective cleaning, creating favorable conditions for periodontal pathogen growth.^18^, ^19^

The current strategy for treating periodontal disease primarily consists of basic periodontal therapy, including scaling and root planning to eliminate supragingival and subgingival plaque and calculus.^20^ However, due to the complex anatomy of the periodontium, the limitations in accessing certain areas during instrument operation, and the propensity for microorganisms to invade and reestablish themselves in periodontal tissues,^21^ the use of antimicrobial agents has become an important supplementary and adjunctive treatment.^22^

Minocycline, known for its broad‐spectrum antibacterial activity against periodontal pathogens, is a commonly used antibiotic in the treatment of periodontal disease.^23^ Recent study suggests that it also has potential for tissue regeneration by inhibiting collagenase and matrix metalloproteinases (MMPs) and by promoting new bone formation.^24^, ^25^ Traditional oral administration methods suffer from drawbacks such as high dosage, low local concentration, difficulty in achieving efficacy, and systemic side effects.^21^ Therefore, local drug delivery has emerged as an effective strategy to enhance local concentration and efficacy while minimizing side effects. In recent years, local delivery of sub‐antimicrobial dose minocycline has become a focus of research.^26^ For instance, Ma et al. have developed minocycline‐loaded nanofiber membranes,^27^ while Zhang et al.^28^ have worked on minocycline‐loaded microspheres. Currently, Periocline® ointment is the most widely used minocycline product on the market.^29^, ^30^ Both in‐development minocycline formulations and clinically utilized medications currently necessitate specialized delivery devices for administration into periodontal pockets. However, formulations designed for application in periodontal pockets often pose difficulties for patients to self‐administer, requiring specialized delivery devices or assistance from healthcare professionals. Additionally, injecting drugs into periodontal pockets can cause swelling, pain, and discomfort, which are not conducive to patient self‐management. Therefore, it is necessary to develop a more convenient, efficient, and comfortable delivery method for adjunctive treatment of periodontitis.

Microneedles (MNs), an innovative drug delivery system featuring microfine needles on a patch, enable precise, painless, and minimally invasive transdermal drug delivery.^31^, ^32^ The targeted and localized delivery capabilities of MNs can enhance drug efficacy, minimize wastage, and reduce systemic side effects.^33^, ^34^, ^35^ Recently, MNs have shown great promise for the delivery of drugs to oral tissues, particularly for treating periodontitis. For example, Zhang et al.^36^ reported a modular MN designed for the controlled release of tetracycline and cytokines, aiming to local immunomodulation and regeneration of periodontal tissue. In addition, Song et al. developed bee sting‐inspired, inflammation‐responsive MNs to deliver metronidazole for the treatment of periodontal disease.^37^ MNs have shown great potential in periodontal drug delivery and the treatment of periodontitis; however, to the best of our knowledge, the MNs for delivering minocycline in treating periodontitis have not yet been studied.

In our study, we introduce a minocycline‐loaded dissolvable microneedle (Mino‐DMN) patch that allows for local and efficient delivery of minocycline to gingiva for the treatment of periodontitis. A two‐step casting micro‐molding process involving vacuum drying and freeze drying is employed to concentrate minocycline in the MNs part and limit its diffusion into the patch backing. The resulting DMN patch features an array of minocycline‐enriched gelatin MNs with a porous HA patch backing. The MNs can penetrate into gingiva with enough mechanical strength and quickly release minocycline into the gingival tissue, ensuring prolonged local residence of the drug and minimizing its loss to saliva. The porous patch backing can rapidly dissolve in saliva, reducing the sensation of foreign objects in the oral cavity and ensuring a more comfortable administration process. In vitro antimicrobial experiments demonstrate that Mino‐DMN exerts potent and prolonged antibacterial effects. In a periodontitis model using Sprague–Dawley rats (SD rats), Mino‐DMN effectively enhances alveolar bone height and volume, suppresses the expression of bone‐resorbing factors, and alleviates inflammatory responses in periodontal tissues. These effects surpass those achieved solely by topically applied minocycline and are comparable to the inconvenient and discomfort administration of Periocline®. Given its ease of use, comfort, and effectiveness, Mino‐DMN emerges as a compelling alternative for adjunctive therapy in periodontal disease, underscoring the substantial potential of MNs in periodontal applications.

## 2 MATERIALS AND METHODS

### 2.1 Materials

All chemical reagents used were of analytical grade. Sodium hyaluronic acid (HA, Mw 100 kDa) was purchased from Bloomage Biotechnology Co., Ltd. (China). Gelatin from porcine skin (type A) was acquired from Sigma‐Aldrich. Minocycline hydrochloride was obtained from Macklin. Polydimethylsiloxane (PDMS, 184 Sylgard) was purchased from Dow Corning. Rat TNF‐α, IL‐1β, IFN‐γ, IL‐10, and TGF‐β1 assay kits were purchased from Solarbio Technology Co., Ltd. (Beijing, China). The reverse transcription kit was procured from Takara (Japan). The RNA extraction kit and the qRT‐PCR assay kit were sourced from ESscience (Shanghai, China). Minocycline Hydrochloride Ointment (Periocline®) was purchased from Sunstar INC (Japan). Unless otherwise specified, all other chemical reagents were provided by Aladdin Biotechnology Co., Ltd. (Shanghai, China).

### 2.2 Animals

Animal experiments were performed in accordance with ethical approval by the Animal Research Ethics Committee of Institute of Hangzhou Institute of Medicine (HIM), Chinese Academy of Sciences (2024R0003). Rats used in this study were purchased from the Zhejiang Laboratory Animal Center. All rats were housed in a sterile environment with conditions maintained at 20–25°C, 30%–70% humidity, and a 12 h light/12 h dark cycle. They had unlimited access to water and food.

### 2.3 Fabrication of the Mino‐DMN patch

A PDMS mold was used to create negative molds for the master metal molds, featuring a 10 × 10 array with 300 μm backing side length, 800 μm spacing, and 1000 μm height. The proposed DMN patches were fabricated by two‐casting micro‐molding. A solution of minocycline hydrochloride (40 mg/mL) and gelatin (150 mg/mL) dissolved in Mili‐Q water was cast onto the PDMS mold. Subsequently, the mold got vacuum degassed in a vacuum chamber at a pressure of 0.1 MPa for 5 min for complete filling of the cavities. After that, any excess solution was removed. The solution then got with vacuum dried at 37°C. For studies requiring fluorescence imaging, minocycline was substituted with Rhodamine 6G or sodium fluorescein during MNs preparation. The patch backing was prepared by dissolving sodium hyaluronate (100 kDa, 150 mg/mL) in Mili‐Q water with sucrose (50 mg/mL), casting the solution onto the mold with tips and degassing it. The mold was then freeze‐dried and the DMN patch peeled off from the mold.

### 2.4 Morphology of Mino‐DMN patch

The general morphology of the fabricated Mino‐DMN patch was observed using the stereo microscope (SZX16, Olympus). The detailed morphology of the Mino‐DMN patch and the micro‐scaled porous structure of the patch backing were imaged by field emission scanning electron microscope (FESEM; JSM‐IT800, JEOL). For porous structure imaging, the Mino‐DMN patch was first immersed in liquid nitrogen and then cut vertically with a scalpel to expose the intact cross section of the patch backing. To visualize the spatial distribution of drug in the DMN patch, general stereo images were acquired using the stereo microscope (SZX16; Olympus), and the detailed images of a single row array of a DMN patch were acquired using an inverted fluorescence microscope (CKX53; Olympus).

### 2.5 Mechanical property of Mino‐DMN patch

The mechanical properties of the Mino‐DMN patches were assessed using a tensile meter (68SC‐05; Instron). Briefly, a vertical force was applied perpendicular to the upward‐facing MN tips fixed on a flat, rigid iron plate. The force was applied by a probe moving downwards at a constant speed of 0.5 mm per minute. The stress‐displacement curve was measured until reaching a displacement of 0.6 mm.

To evaluate the penetration performance of the DMN patch, fresh oral cavities of SD rats were used. Cheek of the rat was carefully dissected using a surgical scalpel and then flattened on a petri dish, ensuring that the mucosal side faced upwards. The DMN patch loaded with Rhodamine 6G was pressed down for 20 s and then gently removed. The microholes created by the DMN patch on the oral mucosa were observed using a stereomicroscope (SZX16; Olympus). For insertion depth analysis, the DMN‐treated cheek tissue was fixed with 4% paraformaldehyde and subsequently sliced using a cryostat microtome (NX70; Thermo Scientific). The prepared samples were then imaged using an inverted fluorescence microscope (CKX53; Olympus).

### 2.6 Drug release in vitro and dissolution behavior of the DMN patch

The drug release study was performed using fluorescein‐loaded DMN patch. Initially, a 5 × 5 DMN patch was immersed in 1 mL of PBS at 37°C in a 24‐well microplate. At designated time intervals, a 50 μL aliquot of the solution was withdrawn to a 96‐well plate for quantification, with 50 μL of PBS immediately replenished. The fluorescence intensity of fluorescein in all samples in the well plate was measured using a microplate reader (TECAN, Spark), followed by the calculation of the cumulative release of fluorescein.

### 2.7 Drug release and degradability of the DMN patch in vivo

In vivo drug release experiments of the DMN patches containing Rhodamine 6G (50 μg) were conducted in SD rats. After intraperitoneal injection of sodium pentobarbital anesthesia, the rats were immobilized on the surgical table, and the gingival mucosa of the upper anterior teeth area was exposed. Subsequently, the gingival surfaces were treated with DMN administration (DMN group) and topical application of Rhodamine 6G (Topical group), respectively. Fluorescence images were acquired at specified times using an in vivo imaging system (IVIS Lumina Series III; PerkinElmer), and Image J software was used to process and analyze fluorescence intensity, with data normalized to the fluorescence intensity measured at 0 minute.

### 2.8 Antibacterial assay

Mino‐DMN's antibacterial properties were assessed using the Kirby‐Bauer disk diffusion method. Resuscitated *Porphyromonas gingivalis* ATCC33277 were cultured on Columbia blood agar plates placed in an anaerobic incubator (90% N_2_, 5% H_2_, 5% CO_2_, 37°C). After 7 days of incubation, a single colony was selected for subculture and amplification (Figure S1A). The blood agar plates were evenly divided into three sections: the blank Control group, the Blank‐DMN group, and Mino‐DMN group (Figure S1B). A bacterial suspension of 10^7^ CFU/mL was prepared, and 100 μL of the suspension was uniformly spread on each plate using a spreader. The blank Control group area receives no drug application. In the Mino‐DMN group area, DMNs loaded with 0.5 μg minocycline are centrally inserted, while in the Blank‐DMN group area, DMNs without drug loading are inserted at the center likewise. The plates were then incubated in an anaerobic incubator for 7 days, and the diameter of the inhibition zones was measured using a caliper.

### 2.9 Establishment of the periodontitis rat model and in vivo study

Forty‐two male SD rats (3 weeks old, weighing 80–100 g) were randomly and evenly divided into the following seven groups: (1) Control group (no establishment of periodontitis model), (2) PD group (periodontitis), (3) UT group (periodontitis without any treatment), (4) Mino‐DMN group (periodontitis treated with DMN loaded with 50 μg minocycline, every other day for a total of 4 doses), (5) Blank‐DMN group (periodontitis treated with DMN loaded without minocycline, every other day for a total of 4 doses), (6) Perio® group (periodontitis treated with 2.5 mg Periocline® ointment, every other day for a total of 4 doses), and (7) Mino‐Topical group (periodontitis treated with 200 μg minocycline topical application, every other day for a total of 4 doses). In essence, rats in Groups 2 to 7 underwent periodontitis induction. After a one‐week acclimatization period in the animal facility, Archimedes antibiotics were administered in the drinking water of rats in Groups 2 to 7 for 4 days, followed by a 3‐day period with water without drugs. The rat periodontitis model was established by ligature placement using 0.1 mm orthodontic stainless wire, along with weekly application of 100 μL of 10^8^ CFU/mL *Porphyromonas gingivalis* suspension to the left maxillary first molar. If the orthodontic wire becomes loose or detached, it should be promptly replaced and re‐ligated. The rats' body weight was recorded, and their gingival index (GI) and tooth mobility (MD) at the ligated site were examined using a periodontal probe during the period. After 4 weeks, the ligature was removed. Rats in Groups 1 and 2 were humanely euthanized, and the left maxillary bones were collected for micro‐CT scanning. Alveolar bone loss (ABL), bone volume fraction (BV/TV), bone surface area ratio (BS/BV), and trabecular thickness (Tb.Th) at the maxillary left first molar ligature site were quantified using CT analysis software. Additionally, histological sections with HE staining were performed to assess the periodontitis modeling conditions.

After the establishment of the model, ligatures at the left maxillary first molars were removed from rats in Groups 3 to 7. Local plaque residues were gently cleaned using a probe and physiological saline. In the Mino‐DMN group, Mino‐DMN patches were firmly pressed to the buccal and palatal sides of the left maxillary first molars for 5 minutes, ensuring that the drug was fully delivered into the gingiva of rats, as well as in the Blank‐DMN group. In the Perio® group, the needle was inserted into the bottom of the periodontal pocket of the upper left first molar of the rats, and the ointment was injected around the root of the tooth, ensuring that the drug fully entered the periodontal pocket. In the Mino‐Topical group, minocycline solution was applied to the gingival sulcus and mucosa of the left maxillary first molar. Keep the mouth open for at least 5 min to avoid rapid drug loss. After the first administration, at the fourth week post‐administration, the rats were euthanized, and the left maxillary bones were collected for further experiments. Using CT analysis software, bone parameters were measured again. Furthermore, three consecutive pathological sections were obtained from each rat and subjected to HE staining. After the extraction of the upper left first molar, periodontal tissue was harvested, and the alveolar bone was gently separated from the surrounding soft tissues, including mucosa and gingiva. qRT‐PCR was employed to analyze the expression levels of Col1a1, RUNX2, OCN, BMP‐2, MMP‐8, CathepsinK, and NFATc1 in the alveolar bone. The primer sequences used for amplification are listed in Table 1. ELISA kits were then utilized to determine the expression levels of TNF‐α, IL‐1β, IFN‐γ, IL‐10, TGF‐β1, and other factors in gingival soft tissue.

**TABLE 1:** Primer sequences used for real‐time PCR.

Gene	Sequence (5′ to 3′)
NFATc1	Forward: CAGCCACGAGGCTATCCTGT
	Reverse: TCTCCCGATGTCTGTCTCCC
MMP‐8	Forward: CAGACACCCTGTCCAACCT
	Reverse: GAATCCTGCGGAAAACTGC
CathepsinK	Forward: TTGACTCTGAAGACGCTTACCC
	Reverse: TCACATTATCACGGTCGCAGTT
COL1A1	Forward: AGGATCGGAACCTTCGCTTC
	Reverse: TGGTACATCAGCCCAAACCC
Runx2	Forward: CGCCTCACAAACCACAG
	Reverse: AATGACTCGGTTGGTCTCGG
OCN	Forward: CAACCCCAATTGTGACGAGC
	Reverse: AACGGTGGTGGCATAGATGC
BMP‐2	Forward: GGGACCCGCTGTCTTCTAGT
	Reverse: TCAACTCAAATTCGCTGAGGAC

### 2.10 Statistical analysis

All experiments included biological replicates, with tissue samples sourced from distinct animals, unless stated otherwise. Statistical analysis was carried out using a two‐tailed Student's *t*‐test or the original one‐way ANOVA. Statistical analysis was conducted using a two‐tailed Student's *t*‐test or the original one‐way ANOVA. *p* values less than 0.05 were considered statistically significant (*p < 0.05, **p < 0.01, ***p < 0.001, ****p < 0.0001, ns = no significant difference). Data analysis was conducted using GraphPad Prism 8.3.

## 3 RESULTS AND DISCUSSION

### 3.1 Fabrication and characterization of the Mino‐DMN patch

**FIGURE 1:**
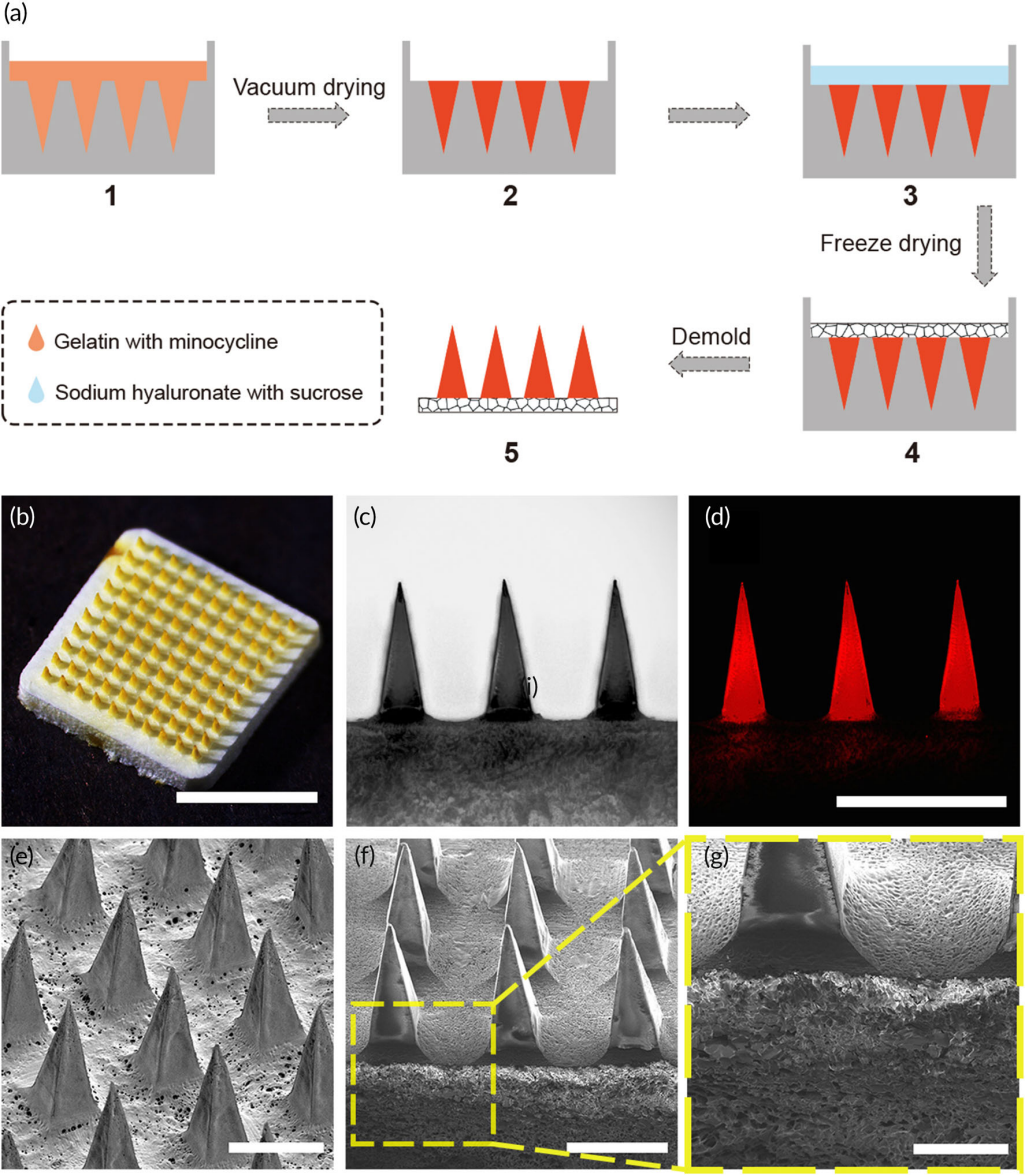
Fabrication and characterization of the Mino‐DMN patch. (a) Schematic of the procedure to fabricate minocycline‐loaded MNs with porous HA patch backing. (b) Stereo bright‐field image of minocycline‐loaded DMN patches with porous HA patch backing. Scale bar, 5 mm. (c) Bright‐field image and (d) fluorescence image of the side view of Rhodamine 6G loaded DMN patch with porous HA patch backing by invert microscopy. Scale bar, 1 mm. (e–g) Scanning electron micrograph images of minocycline‐loaded DMN patch with porous HA patch backing; In f and g, the DMN patch was vertically cut open to expose its patch backing pores. Scale bar, 500 μm, 500 μm and 200 μm for e–g respectively.

The DMN patch loaded with minocycline was fabricated by a two‐step casting micro‐molding process involving vacuum drying and freeze drying (Figure 1a). Briefly, a gelatin solution containing minocycline was cast to cavity of the PDMS mold, and got dried using vacuum at 37°C to form the tip parts. Vacuum drying process helped to concentrate the gelatin and minocycline mixed solution at the tip instead of forming a thin shell on the mold walls while moderate warm condition ensured the fluidity of the solution. Subsequently, the basal part of the mold was filled with a sucrose‐blended HA solution without minocycline. The mold then got freeze‐dried and the DMN patch peeled off to acquire the final product.

Gelatin is an ideal material for Mino‐DMN patch preparation. As the tip material, gelatin does not form an aqueous solution at low temperature, which effectively prevented minocycline diffusion into the HA solution at the patch backing. The freeze‐drying process was utilized to reduce the diffusion of minocycline into the patch backing, and prevent the drug from being washed away by saliva behind the patch backing after administration. The drug was mainly located in the tip part, while the patch backing appears white after freeze drying (Figure 1b). If the patch backing is dried using conventional methods, a large amount of minocycline can be observed diffusing into the patch backing (Figure S2). Quantitatively, compared to the porous backing, a solid backing results in approximately three times greater drug loss, which is unacceptable for the DMN system (Figure S3). The tip‐concentrated structure of the Mino‐DMN patch can be expected to deliver minocycline to the affected area with high efficacy.

To maintain the mechanical strength of the porous backing, sucrose was mixed into the HA solution. Compared to the pure HA solution, the patch backing made from sucrose‐containing HA exhibits a modulus approximately three times higher than that of the pure HA patch backing (Figure S4). This enhancement is attributed to the high mechanical strength of the sucrose crystals.

Observation of the side view of the DMN patches (Figure 1c,d) using an optical microscope revealed that the needle length of the DMN patch array is about 850 μm, and the patch backing width of the DMN patch is about 270 μm, which is smaller than the size of the metal master mold (1000 μm in length and 300 μm in patch backing width). The inconsistency in size was contributed by the shrinkage of the elastic PDMS mold during fabrication. Despite the overall contraction of the DMN patch, according to the SEM images (Figure 1e–g), the DMN patch still maintained a good pyramid shape, with sharp tips suggesting that they can penetrate the gingival smoothly and deliver the drug into the gingival tissues, thus prolonging the retention of the drug compared to simple local application of the drug on the gingival surface.

### 3.2 Mechanical property of the Mino‐DMN patch

**FIGURE 2:**
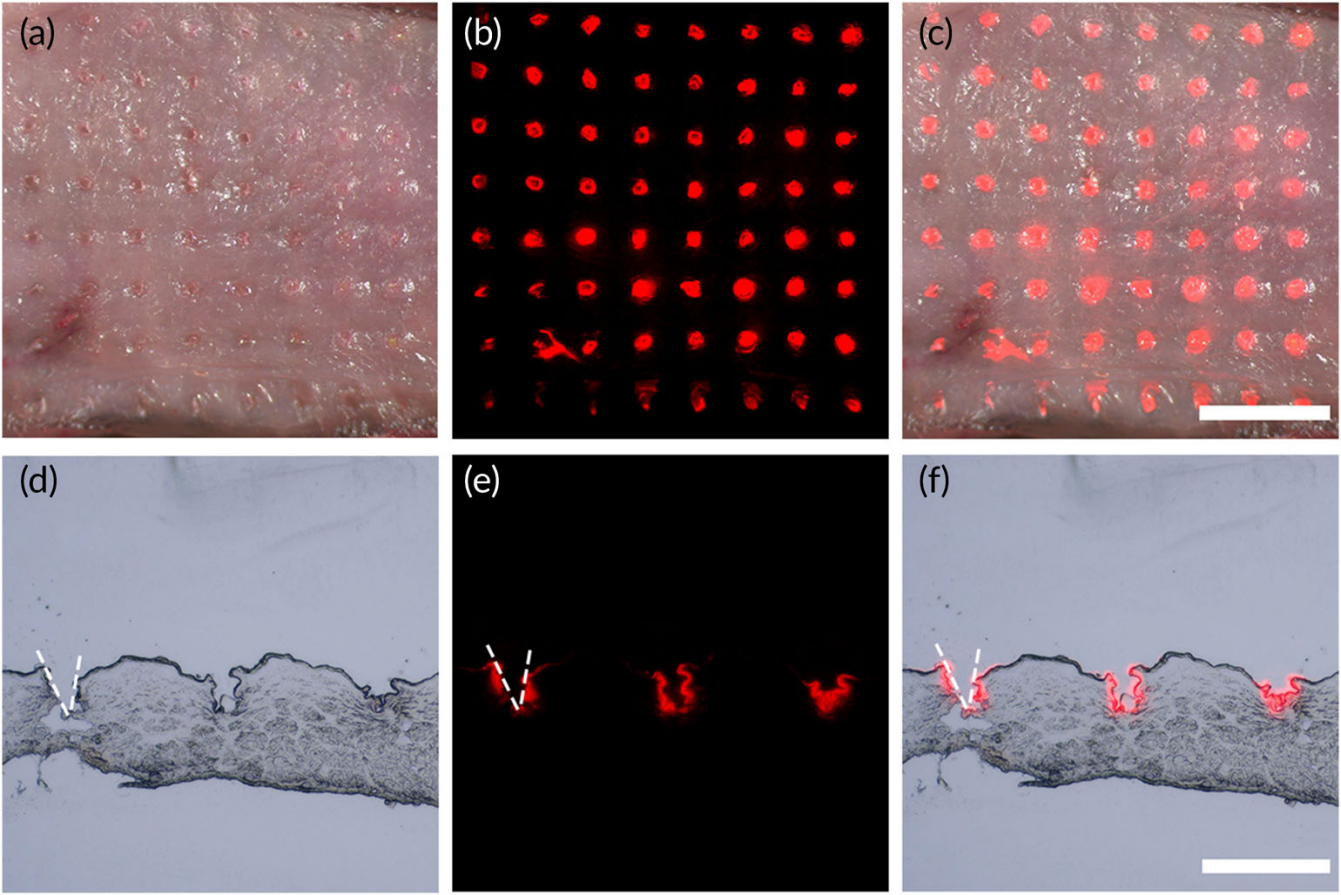
Ex vivo tissue penetration capability of the Mino‐DMN patch. Representative (a) bright‐field, (b) fluorescence, and (c) merge microscopy top view images of SD rat oral mucosa post application of a Rhodamine 6G loaded MN patch ex vivo. Scale bar, 2 mm. Representative (d) bright‐field, (e) fluorescence, and (f) merge microscopy images of frozen sections of SD rat oral mucosa post application of the MN patch loaded with Rhodamine 6G. Scale bar, 500 μm.

A tensile meter was used to evaluate the mechanical properties of the minocycline DMN patches. In simple terms, a force‐sensing probe equipped with mechanical sensors compressed the DMN patch in the vertical direction, and the stress‐displacement curve of the DMN patch during compression was recorded. The results revealed that each tip can exert a force of approximately 0.13 N before fracturing (Figure S5). This force level aligns with the reported MN patches used in gingival administration, demonstrating the capability to effectively penetrate rat gingival tissue.^36^ Furthermore, we explored the penetration ability of Mino‐DMN patches into tissue. We opted to utilize SD rat oral mucosa instead of gingival tissue due to its larger surface area for visualization. By applying pressure, the Mino‐DMN patch easily penetrated the oral mucosa, resulting in the formation of the microholes (10 × 10), which confirmed a 100% penetration rate (Figure 2a–c). Histological examinations clearly showed that the DMN patches were able to penetrate the oral mucosa to a depth ranging from approximately 120–200 μm (Figure 2d). The penetration depth of DMN patch on rat mucosa is about 30% lower than that of MN patches we prepared before on porcine skin.^33^, ^38^ This can be attributed by the comparatively greater softness and elasticity of rat oral mucosa tissue. We also observed that Rhodamine 6G was successfully delivered to the inner wall of the created microholes, with the majority of fluorescence concentrated inside the microholes (Figure 1e,f).

### 3.3 Drug loading and release study of the DMN patch

**FIGURE 3:**
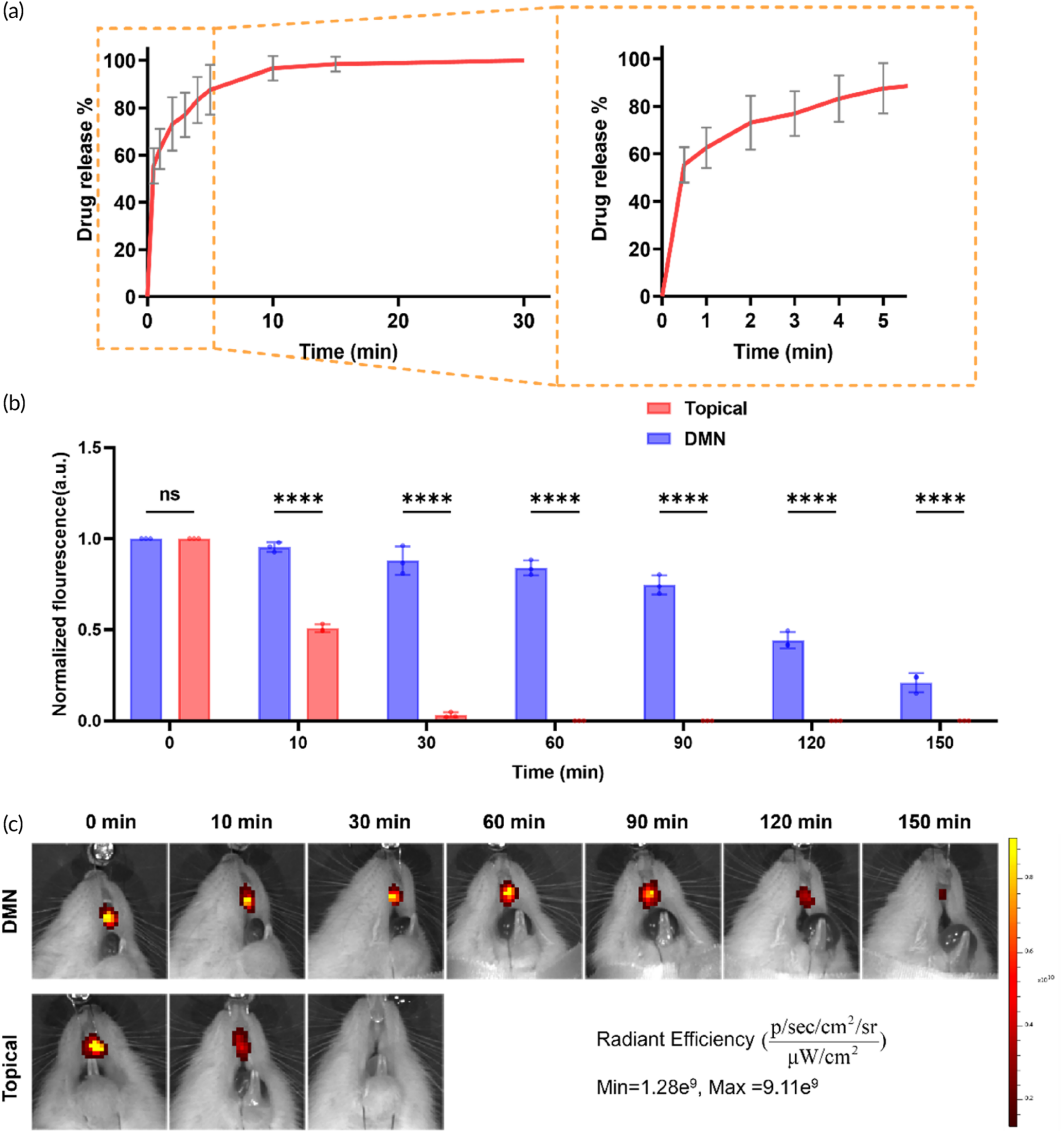
In vitro and in vivo drug release from DMN. (a) In vitro burst release of sodium fluorescein from MN patches in PBS (37°C). Data are presented as mean ± SD. (*n* = 8). (b) Topical application of fluorescein and fluorescein DMN quantified the fluorescence intensity in the gingiva. Data were normalized to the fluorescence intensity at 0 min and presented as mean values. Data are presented as mean ± SD. (*n* = 3). *p < 0.05, **p < 0.01, ***p < 0.001, ****p < 0.0001, ns = no significant difference. (c) Topical application of fluorescein and the fluorescein‐loaded DMN patch for in vivo fluorescence imaging of the gingiva.

Referring to the specifications of the commercially available Periocline® formulation (0.5 g/10 mg) and insights from previous scholarly research on minocycline‐based periodontal formulations,^39^, ^40^, ^41^ we selected a dosage of 50 μg of minocycline per patch. In practical formulation, the actual drug loading achieved was 51 ± 6 μg per patch. In vitro drug release study of the DMN patch involved immersing the patch in PBS at 37°C, while continuously tracking the fluorescence emitted by Rhodamine 6G, a model drug in the DMN patch. The release profile exhibited a remarkably fast drug release from the DMN patch with more than 80% of Rhodamine 6G released within merely 5 min, reaching complete release at 30 minutes (Figure 3a). The rapid‐release profile not only guarantees the immediate onset of the drug but also improves patient compliance with shorter administration durations. In vivo drug release kinetics of the DMN patch in SD rats was also carried out (Figure 3b,c). Experimental findings reveal that following a 30‐min application period, negligible drug residues are detected locally on the gingival surface in the Topical group; conversely, DMN‐mediated drug delivery ensures sustained retention for up to 150 min, effectively mitigating localized drug loss induced by salivary clearance. The porous HA backing can immediately form a viscous protective film after MN administration (Figure S6), preventing the drug from being washed away by saliva.^42^, ^43^ Furthermore, HA also possesses excellent anti‐inflammatory and pro‐repair properties, aiding in alleviating local swelling, pain, and discomfort induced by inflammation, thereby positively impacting the improvement of periodontal disease.^39^, ^44^, ^45^

### 3.4 Delivery of antibiotics by Mino‐DMN and antibacterial effects in vitro

**FIGURE 4:**
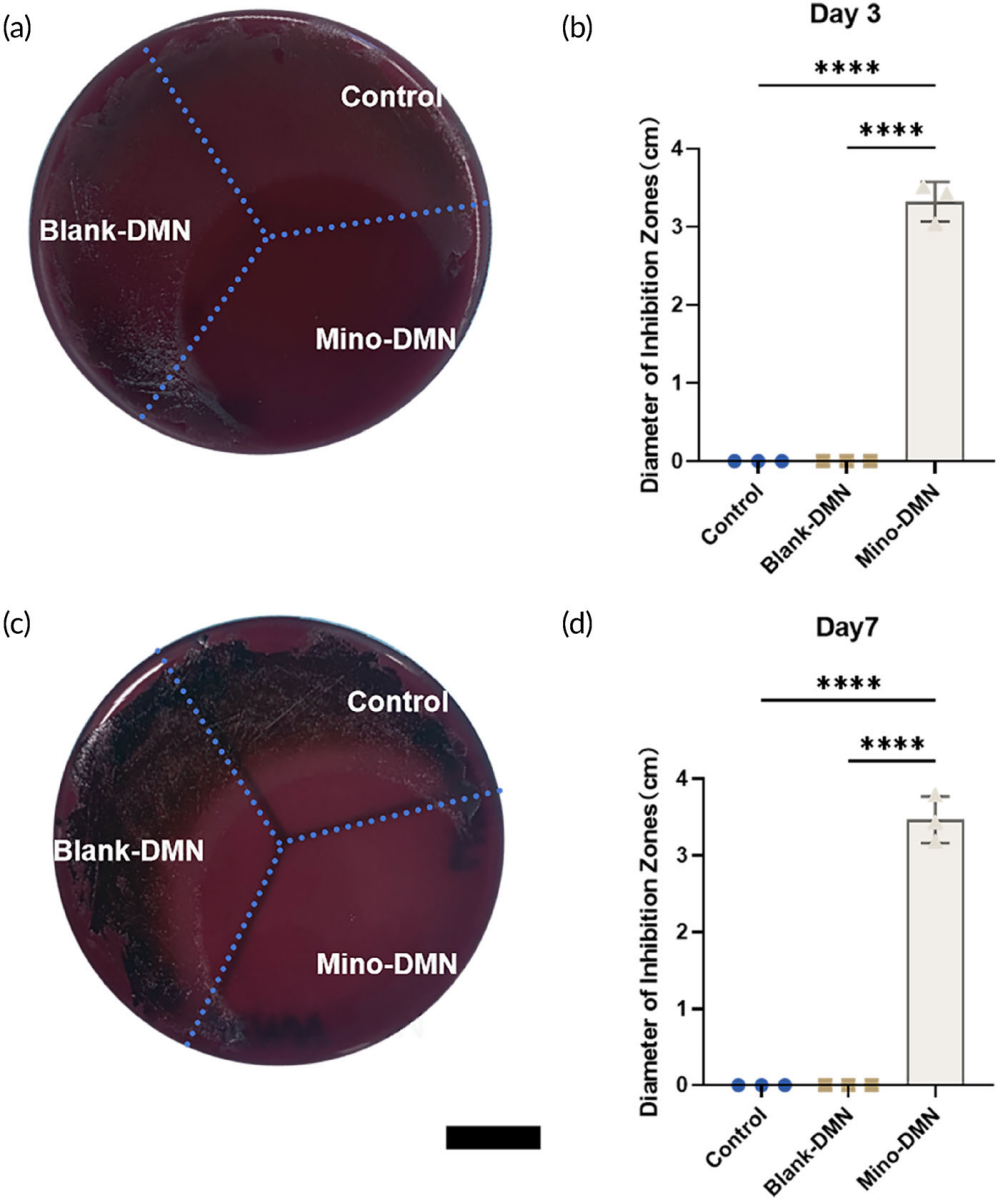
The antimicrobial experiment results indicate that the release of minocycline from Mino‐DMN exhibits antimicrobial effects. (a) After drug inoculation for 2 days. (b) The diameter of the inhibition zone in each group was measured 2 days after drug inoculation. (c) After drug inoculation for 7 days. (d) The diameter of the inhibition zone in each group was measured 7 days after drug inoculation. All data are presented as mean ± SD. (*n* = 3). *p < 0.05, **p < 0.01, ***p < 0.001, ****p < 0.0001, ns = no significant difference. Scale bar, 1 cm.

To assess the antimicrobial efficacy of drug‐loaded patches against *Porphyromonas gingivalis*, we employed the Kirby‐Bauer disk diffusion test. At 2 days post‐inoculation, the Mino‐DMN group exhibited a distinct antibacterial zone on the agar surface with a diameter of 3.32 ± 0.26 cm (Figure 4a,b), and this zone remained significant even after 7 days, with a diameter of 3.47 ± 0.31 cm (Figure 4c,d). In contrast, no antibacterial zones were observed in the Blank‐DMN group or the Control group on either the 2nd or 7th day.

### 3.5 Establishment of the rat periodontitis model

**FIGURE 5:**
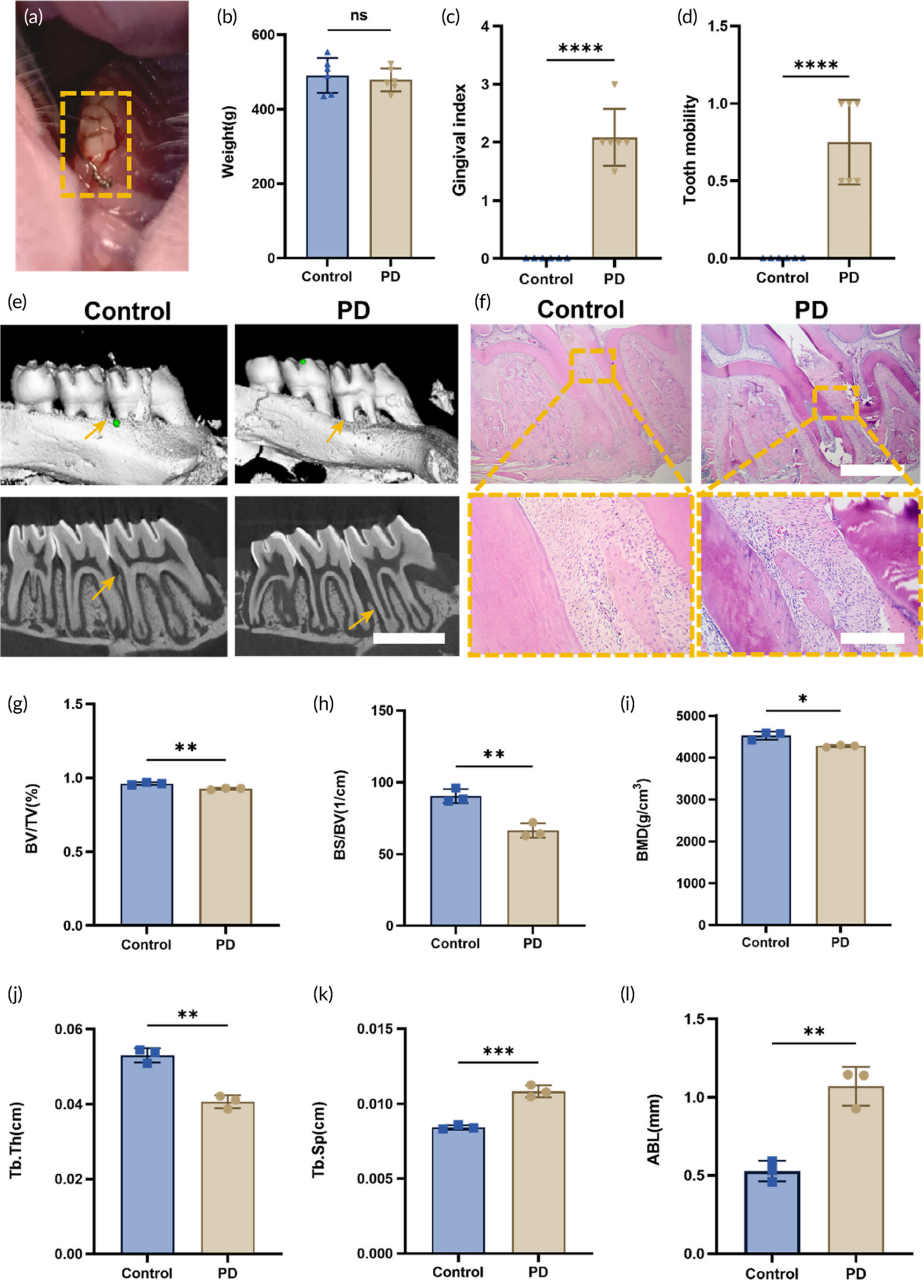
Establishment of periodontitis model in rats. (a) Periodontitis modeling performed on the left upper first molar of rats in the PD group. (b) The changes in body weight of rats in both the Control and PD groups after modeling (*n* = 6). (c) The changes in GI of both the Control and PD groups of rats after modeling (*n* = 6). (d) The changes in MD of both the Control and PD groups of rats after modeling (*n* = 6). (e) The micro‐CT scans and reconstructions of alveolar bone in the Control and PD groups (scale bar, 2 mm), coupled with (f) pathological sections and H&E staining (scale bar, 1 mm and 100 μm), revealed a more pronounced periodontal inflammation in the PD group compared to the Control group. After capturing micro‐CT images, reconstruction and measurements were performed to assess the changes in (g) BV/TV, (h) BS/BV, (i) BMD, (j) Tb.Th, (k) Tb.Sp and (l) ABL after modeling in both the Control and PD groups(*n* = 3). All data are presented as mean ± SD. *p < 0.05, **p < 0.01, ***p < 0.001, ****p < 0.0001, ns = no significant difference.

In order to validate the antibacterial and anti‐inflammatory effects of Mino‐DMN patches on periodontitis, we conducted an in vivo study using a rat periodontitis model. The study involved inducing periodontitis in the upper left first molar of rats through a combination of orthodontic wire ligature and inoculation with *Porphyromonas gingivalis* solution (Figure 5a).

Compared to the Control group, the average body weight was slightly lower in the PD group, but without statistical significance (Figure 5b). After one‐month ligature, the PD group showed darker red gingiva, soft texture, bleeding upon probing, and slight to moderate tooth mobility (Grade 0‐I), indicating more prominent gingival inflammation (Figure 5c,d). According to the three‐dimensional reconstruction images and HE‐stained histological sections (Figure 5e,f), the PD group showed continuous destruction of the gingival epithelium, significant destruction of alveolar bone, with severe absorption at the alveolar crest and even in the root bifurcation area. Additionally, with micro‐CT quantitative measurement, the PD group exhibited significantly reduced BV/TV (Figure 5g), BS/BV (Figure 5h), BMD (Figure 5i), Tb.Th (Figure 5j), and increased Tb.Sp (Figure 5k) and ABL (Figure 5l). These results confirmed the successful establishment of the rat periodontitis model.

### 3.6 In vivo studies of minocycline MNs for periodontitis treatment

#### 3.6.1 Clinical inflammation levels, imaging measurements, and pathological assessment

**FIGURE 6:**
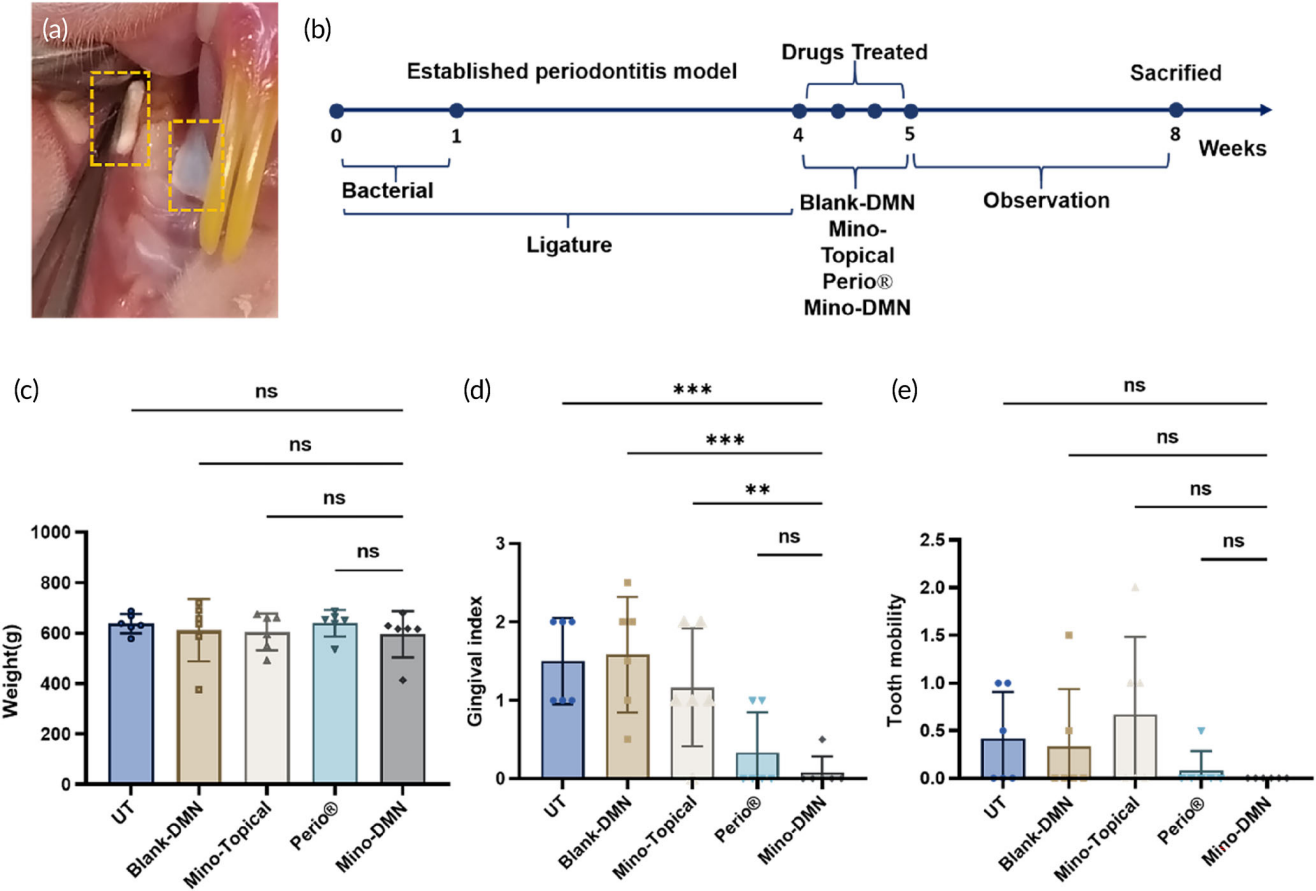
The Clinical Manifestations of Mino‐DMN and Other Drugs in In Vivo Experiments. (a) The MN patches are applied to local periodontal tissues. (b) The schematic diagram of the in vivo experimental procedure. (c) The changes in body weight of each group of rats within 1 month after administration. (d) The changes in GI values of the upper left first molars in each group of rats after administration. The GI scoring criteria are as follows: 0 = Normal Gingiva; 1 = Slight change in color and texture of the gums, slight swelling, but no bleeding on probing; 2 = Redness, swelling, and glazing of the gums, bleeding on probing; 3 = Marked redness and swelling, ulceration, tendency for spontaneous bleeding or profuse bleeding on probing. (e) The changes in MD values of the upper left first molars in each group of rats after administration. The MD scoring criteria are as follows: 0 = Physiological mobility within the normal range; 1 = Mobility is present only in the buccolingual (cheek to tongue) direction; 2 = Mobility is present in both the buccolingual and mesiodistal (toward the adjacent teeth) directions; 3 = Mobility is present in both the buccolingual and mesiodistal directions, and there is also vertical displacement. Data are presented as mean ± SD. (*n* = 6). *p < 0.05, **p < 0.01, ***p < 0.001, ****p < 0.0001, ns = no significant difference.

In order to assess the inhibitory effects of Mino‐DMN patches on periodontitis, rats with successfully induced periodontitis were employed. The study involved the application of Mino‐DMN patches, Blank‐DMN patches, a commercially available product Periocline® ointment (2% Minocycline ointment), and a 2% Minocycline solution to the gingival area surrounding the upper left first molars of the rats. After the removal of orthodontic ligatures from the upper left first molars, meticulous cleaning of dental surfaces was carried out using probes and cotton to eliminate residual food debris. DMN patches were then carefully placed on the palatal and buccal mucosa of the teeth for 5 min (Figure 6a). Gentle pressure was applied to facilitate MN penetration into the mucosa, and the patches dissolved with the drug fully delivering into the gingiva, signifying the completion of drug administration. The entire experimental procedure is illustrated in Figure 6b. After one month of treatment, there were no significant differences in body weight among the rat groups, suggesting no notable systemic toxicity from local drug application (Figure 6c). Compared to the UT, Blank‐DMN, and Mino‐Topical groups, the Mino‐DMN group showed a significant reduction in GI, indicating effective improvement in gingival inflammation with Mino‐DMN usage (Figure 6d). Although the MD values decreased compared to one month prior, the differences among the groups were not statistically significant when compared to UT, Blank‐DMN, and Mino‐Topical groups (Figure 6e). This could be attributed to the relatively mild periodontal lesions in rats, resulting in less obvious tooth mobility.

**FIGURE 7:**
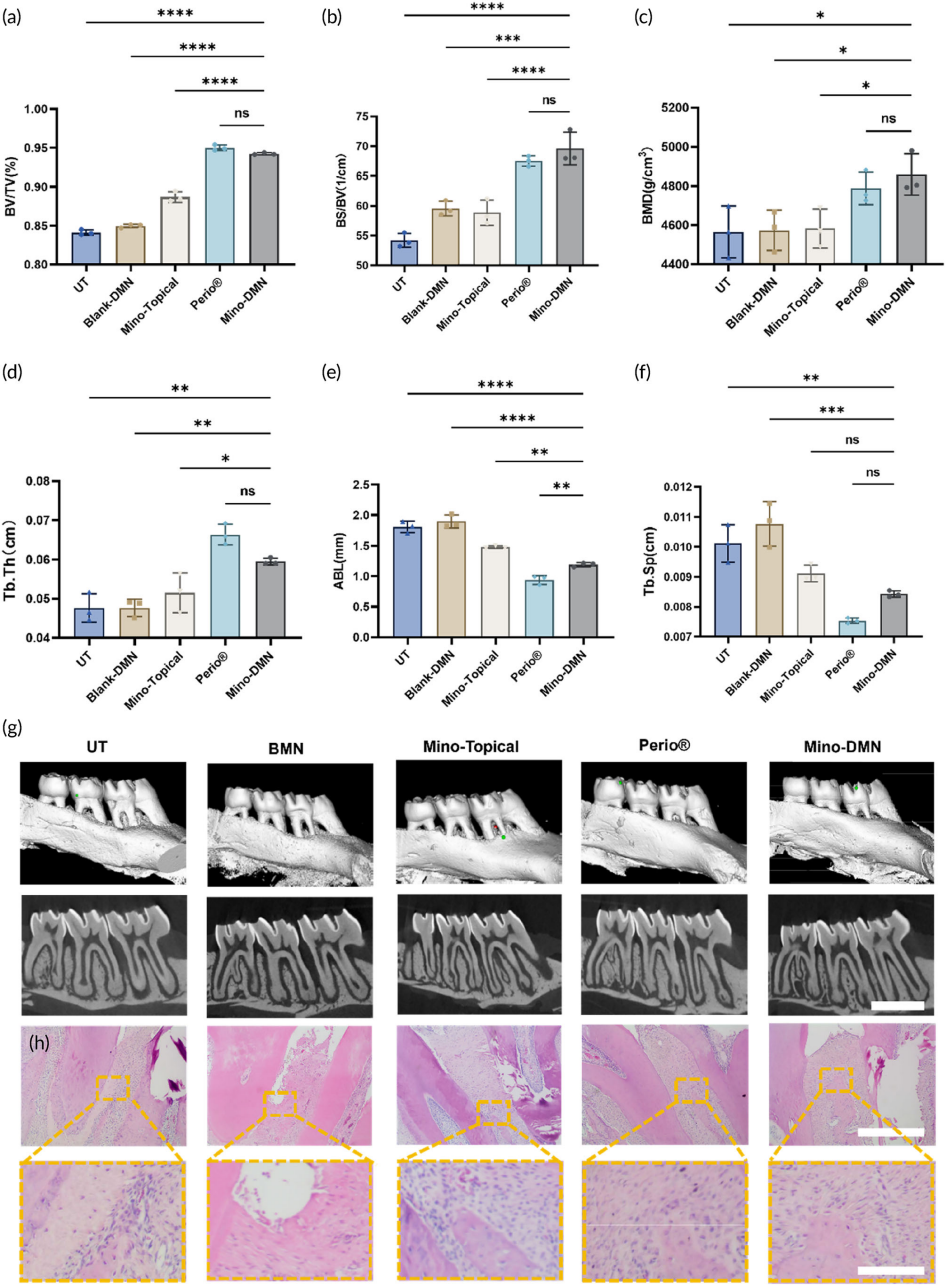
The imaging measurements and pathological manifestations of Mino‐DMN and other drugs in in vivo experiments. After the first time of administration for 4 weeks, micro‐CT scanning and reconstruction were performed on the left upper jawbone of rats. (a) BV/TV, (b) BS/BV, (c) BMD, (d) Tb.Th, (e) ABL, and (f) Tb.Sp were measured and compared for analysis. (g) The left upper jawbones of rats in each group underwent micro‐CT scanning and three‐dimensional reconstruction. Scale bar, 2 mm. (h) Local periodontal inflammation was observed through pathological sections stained with HE. Scale bar, 500 μm and 100 μm. Data are presented as mean ± SD. (*n* = 3). *p < 0.05, **p < 0.01, ***p < 0.001, ****p < 0.0001, ns = no significant difference.

One month after administration, the Mino‐DMN group showed significant reductions in ABL and Tb.Sp values, along with notable increases in Tb.Th, BV/TV, BS/BV, and BMD, indicating effective prevention of alveolar bone loss by Mino‐DMN. Compared to the Mino‐Topical group, Mino‐DMN exhibited reduced ABL and increased BV/TV, BS/BV, BMD, and Tb.Th, suggesting that topical minocycline application inhibits alveolar bone loss, but Mino‐DMN is notably more effective. Compared to the Perio® group, ABL values were significantly higher in the Mino‐DMN group, with no differences observed in other indicators, indicating that the efficacy of Mino‐DMN in preventing alveolar bone loss is comparable to that of Periocline® ointment. Considering that Periocline® ointment is administered long‐term through periodontal pockets, while Mino‐DMN is rapidly delivered through the gingival surface, along with potential biases in animal experiments, this disparity of ABL can be understood (Figure 7a–f).

Through 3D reconstructed images (Figure 7g) and pathological sections (Figure 7h), we observed slight absorption between the first and second molars in the Mino‐DMN and Perio® groups, with minimal absorption in the root bifurcation area. Gingival epithelium showed good continuity, indicating mild inflammation. In the Mino‐Topical group, significant absorption in the alveolar ridge and root bifurcation area suggested severe inflammation, with disordered deep periodontal fibers. The UT and Blank‐DMN groups exhibited severe absorption in the same region, poor epithelial continuity, and more severe attachment loss. Periodontitis severity ranked as follows: Mino‐DMN and Perio® groups < Mino‐Topical group < UT and Blank‐DMN groups.

#### 3.6.2 Molecular‐level assessment of local soft tissue inflammation and bone tissue destruction in periodontal tissues

To assess minocycline MNs' molecular anti‐inflammatory effects, we analyzed periodontal tissues from the left upper first molars in each rat group. ELISA quantified pro‐inflammatory and anti‐inflammatory factor expression in soft tissues, while q‐PCR measured mRNA levels of osteogenic and osteoclastogenic genes in alveolar bone.

**FIGURE 8:**
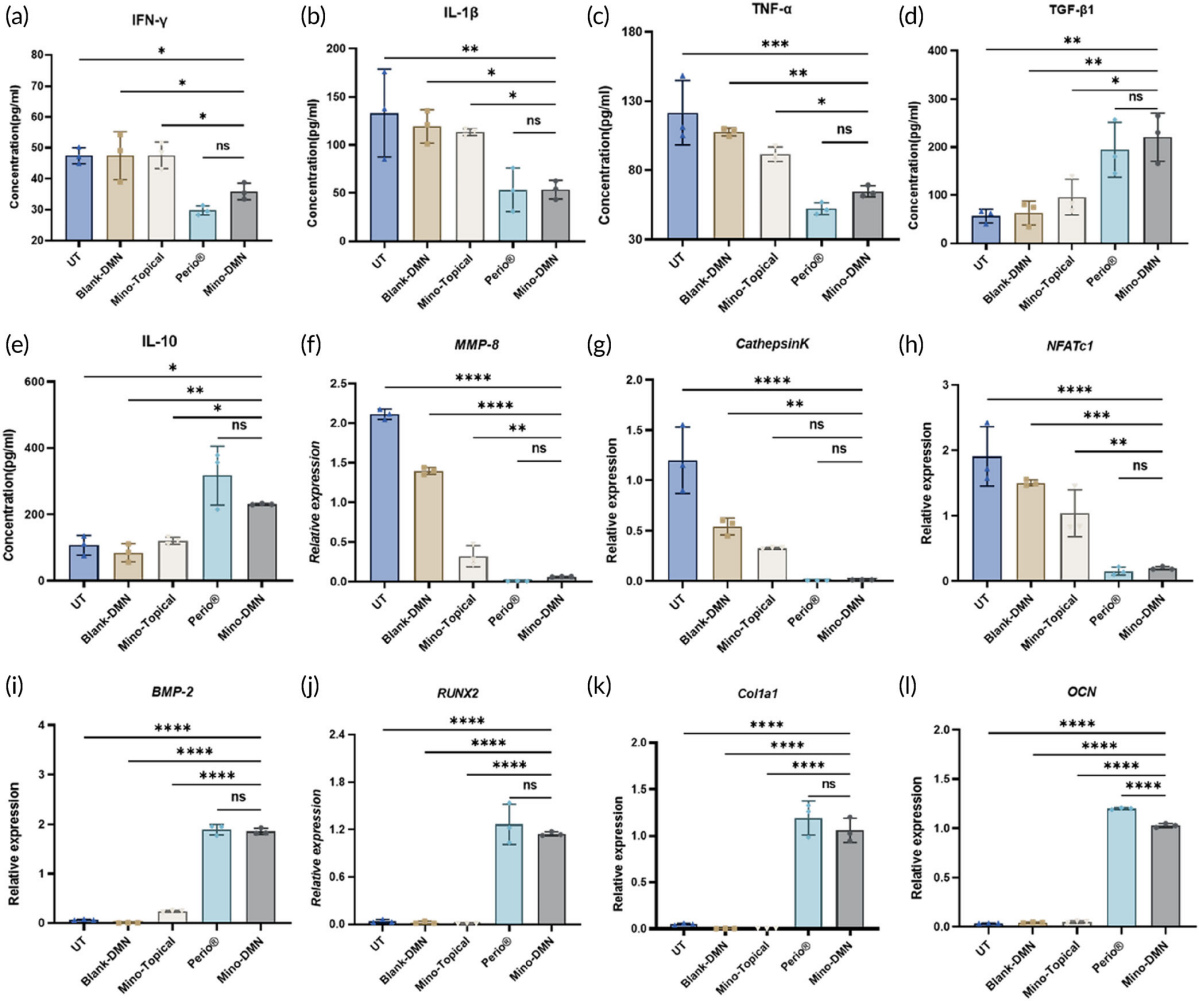
In Vivo Experiments Validate the Anti‐inflammatory Effects and Osteogenic Promotion of Mino‐DMN. The expression of (a) IFN‐ γ, (b) IL‐1β, (c) TNF‐α, (d) TGF‐β1, and (e) IL‐10 in periodontal tissues of rats in each group was evaluated using the ELISA method. The expression levels of (f) MMP‐8, (g) Cathepsin K, (h) NFATc1, (i) BMP‐2, (j) RUNX2, (k) COL1A1, and (l) OCN in the alveolar bone of rats in each group were analyzed using q‐PCR method. Data are presented as mean ± SD. (*n* = 3). *p < 0.05, **p < 0.01, ***p < 0.001, ****p < 0.0001, ns = no significant difference.

In periodontal soft tissues, the Mino‐DMN group demonstrated significantly reduced pro‐inflammatory factors (IFN‐γ, IL‐1β, and TNF‐α) and markedly increased anti‐inflammatory factors (TGF‐β1 and IL‐10) compared to UT, Blank‐DMN, and Mino‐Topical groups. Compared to the Perio® group, the Mino‐DMN group showed no significant difference (Figure 8a–e). The anti‐inflammatory attributes of minocycline in periodontal tissues enjoy broad recognition.^25^ Minocycline has been demonstrated to inhibit local inflammatory responses by downregulating the production of inflammatory factors such as TNF‐α.^46^ DMN ensures accurate drug delivery deep into the tissues, thereby extending the residence time of trace drugs in the periodontium effectively. This substantially enhances its efficacy while minimizing drug wastage. These results underscore the strong anti‐inflammatory properties of Mino‐DMN, highlighting their potent efficacy in inflammation inhibition.

In alveolar bone, the Mino‐DMN group exhibited significantly higher expression levels of osteogenic genes (COL1A1, OCN, RUNX2, and BMP‐2) compared to UT, Blank‐DMN, and Mino‐Topical groups. Additionally, osteoclastogenic genes like NFATc1 and MMP‐2 showed notably lower expression. CathepsinK also significantly decreased compared to UT and Blank‐DMN groups (Figure 8f–l). Compared to the Perio® group, except for slightly lower OCN mRNA expression, other values showed no significant differences. These results, consistent with micro‐CT scans and pathological sections, affirm minocycline MNs' robust inhibitory effect on bone resorption, promotion of bone metabolism, acceleration of new bone formation, and facilitation of periodontal tissue regeneration. Previous studies have shown that minocycline can effectively inhibit MMPs, prevent the destruction of periodontal connective tissue, and block alveolar bone resorption.^46^ At the same time, it can upregulate the expression level of RUNX2, OCN, and other related osteogenic genes, promote the differentiation and mineralization of osteoblasts, and promote alveolar bone repair.^47^ Therapeutic effect of Mino‐DMN surpasses topical minocycline application and approaches, if not matches, the effectiveness of the commercial periodontal medication Periocline® ointment. Despite the prolonged‐release features of Periocline® ointment, research indicates that minocycline concentration within the periodontal pocket can decrease to only 19.7% of its initial level within 3 hours post‐administration, with rapid release still observed within 7 h.^48^ While Mino‐DMN functions as a fast‐release formulation, its unique design ensures precise delivery of minute medication doses and sustained drug presence in periodontal tissues. This may contribute to its efficacy in treating periodontitis, akin to Periocline® ointment. Considering its ease and comfort of administration, Mino‐DMN emerges as a promising alternative for adjunctive periodontal therapy. Although the Mino‐DMN system was conceptualized using minocycline as a model drug, its adaptability extends to other drug types, bioactive molecules, and sustained‐release formulations.

In summary, the application of MN patches in the realm of periodontics holds immense promise. Our Mino‐DMN system represents a significant stride forward. Future advancements may encompass synergizing diverse drugs or bioactive molecules and developing sustained‐release formulations tailored for MN loading. This approach, as substantiated by the present study, offers boundless potential and auspicious prospects for the field.

## 4 CONCLUSIONS

This study developed a Mino‐DMN patch for adjunctive therapy in periodontitis. Employing a two‐step casting micro‐molding process involving vacuum drying and freeze drying, minocycline is concentrated in the MN portion, combined with a porous HA patch backing. MNs can penetrate the gingiva with sufficient mechanical strength, swiftly releasing minocycline into the gingival tissue, ensuring prolonged local drug residence and minimizing saliva loss. Mino‐DMN exhibits persistent antimicrobial effects and effectively enhances alveolar bone height and volume, alleviating inflammation in periodontal tissues. These effects surpass those achieved solely by topically applied minocycline and are comparable to the inconvenient and discomfort administration of Periocline®. Due to its user‐friendly nature, comfort, and effectiveness, Mino‐DMN presents a fresh approach to adjunctive therapy for periodontal disease, showcasing the significant potential of MNs in oral treatments.

## AUTHOR CONTRIBUTIONS

**Huimin Li:** Data curation; formal analysis; investigation; methodology; software; visualization; writing – original draft. **Xueyu Wen:** Data curation; formal analysis; investigation; methodology; software; visualization; writing – original draft. **Xinyi Gong:** Data curation; investigation; methodology; resources; software; visualization. **Yange Wu:** Investigation; supervision; writing – review and editing. **Puxuan Zhao:** Investigation; software; validation. **Yun Zhang:** Investigation; methodology; validation. **Zhuomin Sha:** Investigation; validation. **Hao Chang:** Conceptualization; funding acquisition; project administration; resources; software; writing – original draft; writing – review and editing. **Xuepeng Chen:** Conceptualization; funding acquisition; project administration; resources; software; writing – review and editing.

## CONFLICT OF INTEREST STATEMENT

The authors confirm that this article content has no conflict of interest.

### PEER REVIEW

The peer review history for this article is available at https://www.webofscience.com/api/gateway/wos/peer-review/10.1002/btm2.10730.

## Supporting information


Data S1

## Data Availability

The data for this study are available within the article, with additional data available in the Supporting Information.
