# Supplementary material for: Facile minocycline deployment in gingiva using a dissolvable microneedle patch for the adjunctive treatment of periodontal disease
Source: Bioeng Transl Med. 2024 Oct 20;10(2):e10730. doi: 10.1002/btm2.10730 (PMC11883109; doi:10.1002/btm2.10730)
Supplement: Supplementary file 1 — Data S1 [file BTM2-10-e10730-s001.docx]

**Supporting information**


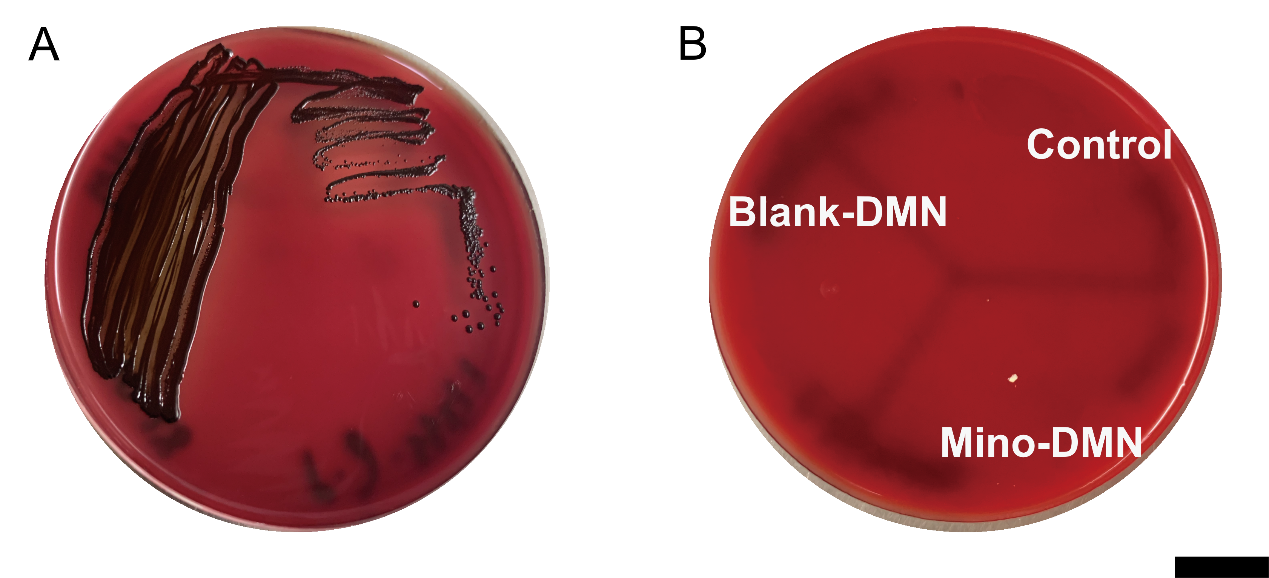


Fig. S1. Antimicrobial experiments. (A) Isolate and select single colonies of *Porphyromonas gingivalis* for amplification culture, intended for subsequent antimicrobial experiments. (B) Inoculate Mino-DMN and Blank-DMN on Columbia blood agar plates. Scale bar, 1cm.


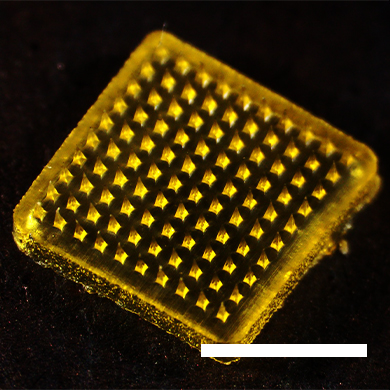


Fig. S2. Stereo bright-field image of Mino-DMN patch with solid HA base. Scale bar, 5mm.

*Spatial distribution of fluorescein in DMNs*

The microneedle patches with porous backing (porous DMNs) were fabricated following the protocol outlined in Section 2.3 of the main text. Fluorescein served as the model drug in the tips of these microneedles. In contrast to porous DMNs, the microneedle patches featuring a dissolvable solid backing (solid DMNs) were produced using an air-drying technique, as opposed to freeze-drying for porous MNs.

To investigate the drug distribution within the DMNs, the tips of the DMN patches were excised using a scalpel and carefully collected. The excised tips and the remaining patch backings were dissolved in 1 mL of PBS respectively. The fluorescence intensity of the solutions was then quantified using a microplate reader, facilitating the determination of the drug concentration and amount.

The proportion of the drug that diffused into the patch backing decreased significantly from 57% to 21% when switching from a solid to a porous backing.


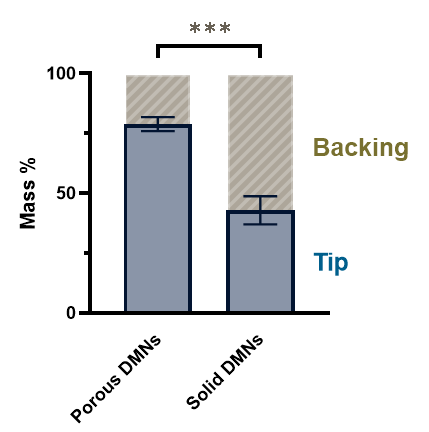


Fig S3. Spatial distribution of fluorescein in DMNs.

*Mechanical Property of DMN patch backing*

Specimens were prepared for a mechanical test. Two types of casting solutions were employed: HA (150 mg/mL) mixed with sucrose (50 mg/mL), and HA (200 mg/mL) alone. These solutions were cast into molds, frozen, demolded, and freeze-dried. The specimen dimensions were 11 mm × 11 mm × 1 mm. Mechanical properties were assessed using a tensile meter, following the method described in Section 2.5 of the main text.

The results revealed that the modulus of the sucrose-mixed HA backing (4.27 ± 0.10 MPa) was significantly higher than that of the pure HA backing (1.45 ± 0.09 MPa), effectively preventing deformation of the MN patches during administration.


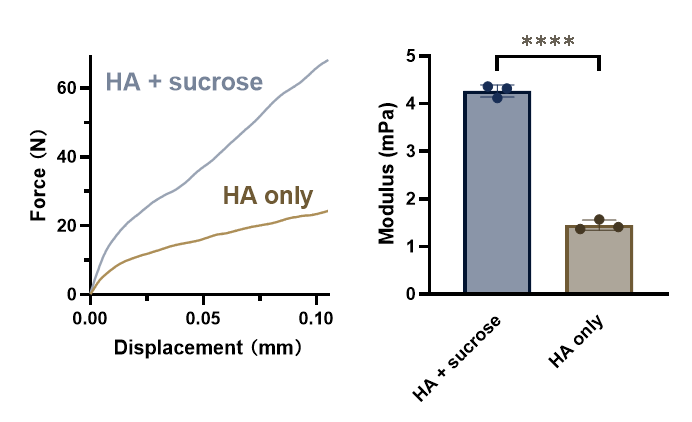


Fig S4. (Left) Stress curve plotted against displacement of specimens, and (Right) Compressive modulus of the specimens.


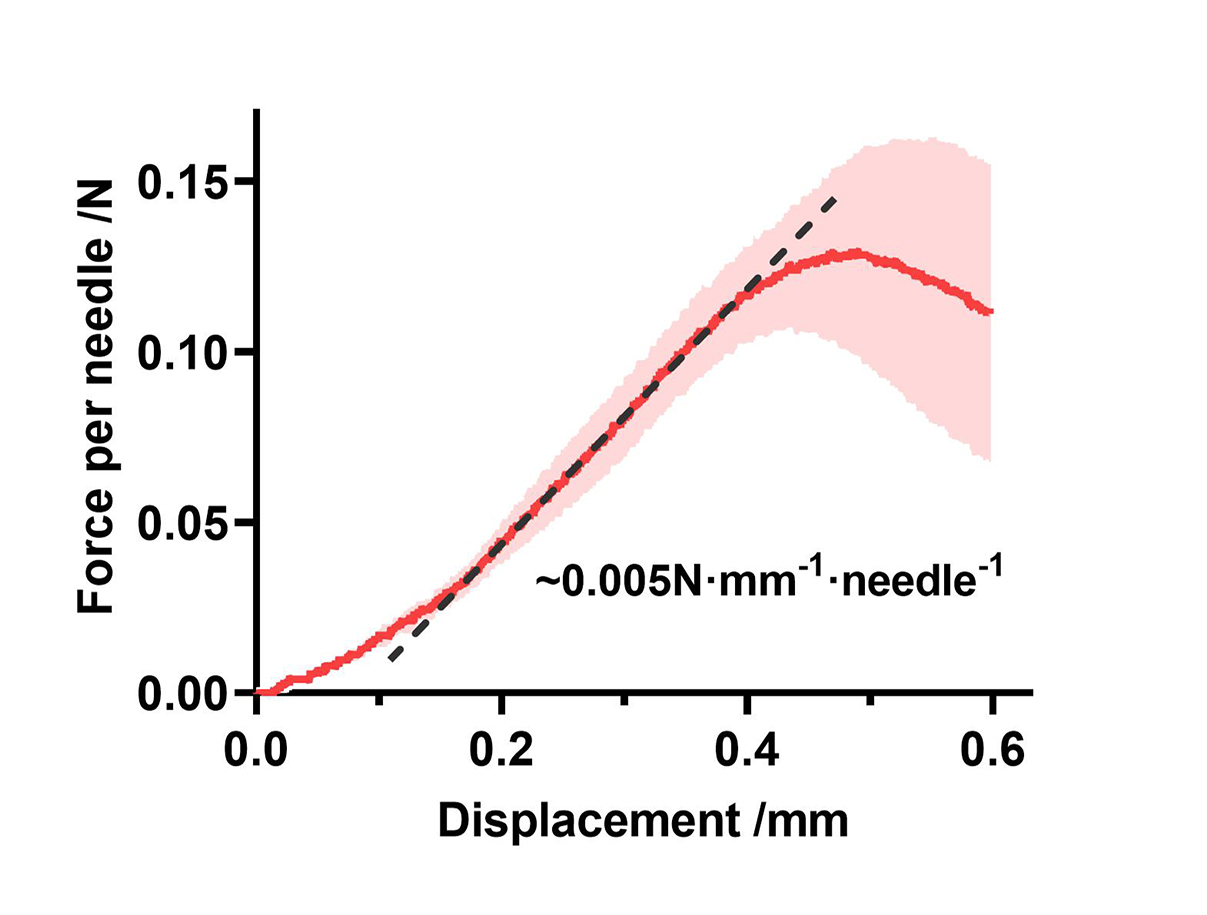


Fig. S5. Mechanical compression tests of the *Mino-DMN patch*. Data are presented as mean ± s.d. (n=10).


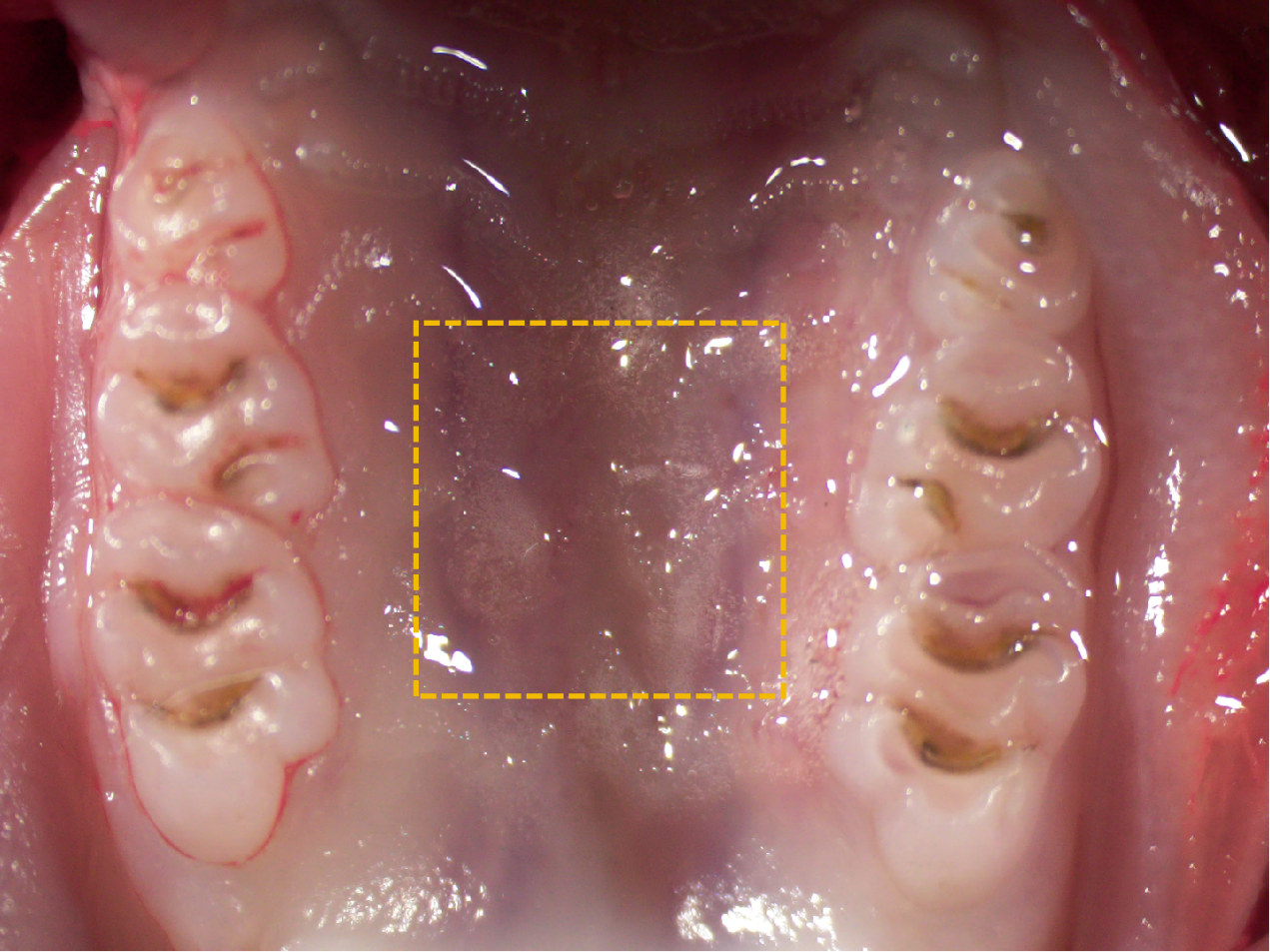


Fig. S6. Upon administration of Mino-DMN, the porous HA backing forms an immediate adhesive protective film.
